# LOWER SOCIOECONOMIC STATUS PREDICTS NEGATIVE PREFERENCES FOR PALLIATIVE CARE

**DOI:** 10.1093/geroni/igad104.1650

**Published:** 2023-12-21

**Authors:** Molly Nowels, Elissa Kozlov, David Nowels, Paul Duberstein

**Affiliations:** Rutgers School of Public Health, Piscataway, New Jersey, United States; Rutgers School of Public Health, Piscataway, New Jersey, United States; University of Colorado School of Medicine, Aurora, Colorado, United States; Rutgers School of Public Health, Piscataway, New Jersey, United States

## Abstract

With an aging US population, more people than ever live with serious illnesses. Although palliative care (PC) can improve outcomes in serious illness, there are inequities in PC utilization. People with low socioeconomic status (SES), men, and Black and Hispanic people are less likely to receive and benefit from PC services. Despite these established demographic differences in PC utilization, there is a dearth of relevant survey research on preferences for PC in the general population. To address this gap, we surveyed a random sample of 1,500 NJ adults. Respondents were given a brief definition of PC and asked to indicate how likely they would be to schedule, attend, and routinely attend PC visit(s) if they were diagnosed with a serious illness. Predictors included in logistic regression modeling were SES indicators (income, educational attainment, insurance status, employment status), gender, and race/ethnicity. Data were weighted to be representative of the population of NJ. Modeling results revealed that lower income and lower educational attainment were associated with significantly lower odds of endorsing willingness to schedule, attend, and routinely attend PC visits in the event that one would become seriously ill. Unexpectedly, there were no gender or race/ethnicity differences in preferences for PC. These findings highlight the importance of public health education for what PC is and its benefits for an aging population, especially among those with lower SES. Future research efforts are needed to understand discrepancies in reported PC preferences versus real-world PC utilization for men and Black and Hispanic individuals.

